# Transcriptome of the Caribbean stony coral *Porites astreoides* from three developmental stages

**DOI:** 10.1186/s13742-016-0138-1

**Published:** 2016-08-02

**Authors:** Tamer A. Mansour, Joshua J. C. Rosenthal, C. Titus Brown, Loretta M. Roberson

**Affiliations:** 1 Department of Population Health and Reproduction, University of California, Davis, California USA; 2 Department of Clinical Pathology, College of Medicine, Mansoura University, Mansoura, Egypt; 3 Marine Biological Laboratory, Woods Hole, Massachusetts USA; 4 Department of Environmental Science, University of Puerto Rico Río Piedras, San Juan, Puerto Rico USA; 5 Institute of Neurobiology, University of Puerto Rico Medical Sciences Campus, San Juan, Puerto Rico USA

**Keywords:** *Porites astreoides*, Calcification, Biomineralization, Coral, *Symbiodinium*, Dinoflagellate, Zooxanthellae, Symbiosis, Swimming larvae, Larval settlement

## Abstract

**Background:**

*Porites astreoides* is a ubiquitous species of coral on modern Caribbean reefs that is resistant to increasing temperatures, overfishing, and other anthropogenic impacts that have threatened most other coral species. We assembled and annotated a transcriptome from this coral using Illumina sequences from three different developmental stages collected over several years: free-swimming larvae, newly settled larvae, and adults (>10 cm in diameter). This resource will aid understanding of coral calcification, larval settlement, and host–symbiont interactions.

**Findings:**

A *de novo* transcriptome for the *P. astreoides* holobiont (coral plus algal symbiont) was assembled using 594 Mbp of raw Illumina sequencing data generated from five age-specific cDNA libraries. The new transcriptome consists of 867 255 transcript elements with an average length of 685 bases. The isolated *P. astreoides* assembly consists of 129 718 transcript elements with an average length of 811 bases, and the isolated *Symbiodinium sp*. assembly had 186 177 transcript elements with an average length of 1105 bases.

**Conclusions:**

This contribution to coral transcriptome data provides a valuable resource for researchers studying the ontogeny of gene expression patterns within both the coral and its dinoflagellate symbiont.

## Data description

### Background

With an increasing focus on threats such as climate change in recent years, there is a growing body of research on the mechanisms underlying coral calcification, and coral response to environmental change [1–6]. Evidence suggests that corals regulate the movement of ions such as bicarbonate, calcium, and hydrogen to facilitate calcification [1, 2, 6, 7], and that some species are more tolerant of changes in their environment [8, 9], yet the mechanisms behind these important processes and their molecular components are unknown. In particular, details of how the symbiotic dinoflagellates (zooxanthellae) enhance calcification and their role in skeleton formation have not been identified to date. A library of gene transcripts from key developmental stages such as settlement can provide valuable information about which genes are important for processes that are turned on at a particular stage, in this case at the onset of calcification. Previous studies have focused individually on the adult stage [3, 5, 10] or the larval stage [11]; few have examined the holobiont, particularly during the early developmental stages [5, 12–14].

As a model, we used the species *Porites astreoides*, a rapidly growing stony coral that is ubiquitous in the Caribbean and relatively tolerant to anthropogenic stresses [15, 16]. Thus, this species may provide insight into changes at the molecular level that have allowed it to resist or acclimate to environmental change. Additionally, the availability of brooded, rapidly settling symbiotic larvae that lack a skeleton [9, 17–19] make this an ideal species for studying early events in the development of the machinery used in calcification, and the role of symbionts in skeleton formation. Although 454 sequence data are already available for *Acropora millepora* larval stages [11], here we present the first transcriptomes for a coral holobiont at multiple life stages with much higher depth and breadth of coverage than previously published *Porites* transcriptomes [3, 11].

Here we used deep sequencing to build a combined pre-/post-settling larval and adult coral metatranscriptome, which includes the zooxanthellae symbionts. The addition of early life history stages and the algal symbiont to currently available assemblies will allow events important to settlement and calcification to be studied, as well as how they relate to simultaneous changes in the holobiont’s gene expression [12, 20]. To aid in the capture of rare holobiont transcripts, including those from organisms besides coral and zooxanthellae (e.g., bacteria [21]), we used relaxed assembly parameters. Though this can potentially include some erroneous assemblies, it is optimized for potentially low abundance organisms. We have included the isolated coral and zooxanthellae transcriptomes through homology filtering to aid in the identification of genes expressed exclusively in the animal or plant components of the holobiont. The annotated transcriptomes presented here should therefore provide a valuable resource for researchers interested in coral calcification, larval settlement, host–symbiont interactions, and development of the complex holobiont.

### Samples

**Fig. 1:**
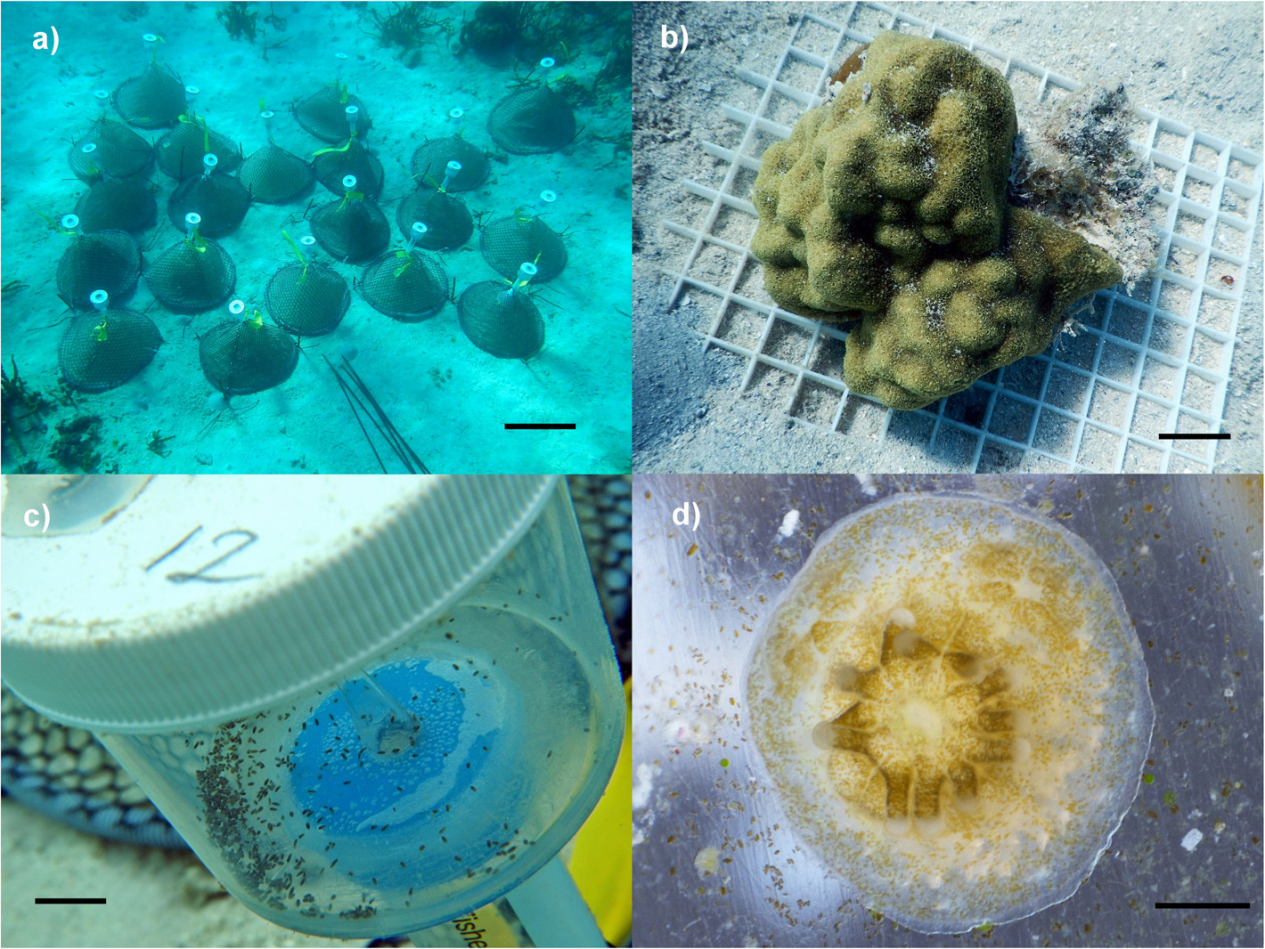
*Porites astreoides* field sample collection. **a** Traps used in the collection of coral larvae. Scale bar = 40 cm. **b** Adult *P. astreoides* colonies used in traps. Scale bar = 3 cm. **c** Swimming larvae collected in trap. Scale bar = 2 cm. **d** Five-day old settled larvae. Scale bar = 1 mm

All corals were collected from El Mario Cay, La Parguera, Puerto Rico (N 17.95258, W 67.0563) in 2012, 2013 and 2014 under permits issued by the Puerto Rico Departamento de Recursos Naturales y Ambientales (Department of Natural and Environmental Resources; DRNA), numbers 2012-IC-051 and #2014-IC-075. Cone traps, developed by Wade Cooper of the Fish and Wildlife Research Institute, Florida (Fig. 1a), were placed and retrieved by SCUBA divers to collect larvae from adult *P. astreoides* colonies in 2012 and 2014. During the time of maximum larval release (April–July), ten healthy adult colonies between 10 and 25 cm in diameter were removed from the reef and placed on racks in an adjacent sandy area to facilitate anchoring the traps, and to minimize damage to the reef (Fig. 1b). Actively swimming planula larvae were collected from each trap the morning after trap placement (Fig. 1c), transported to the Magueyes Island Marine Laboratory in a large cooler, and immediately processed for sampling. Larvae from each trap were kept separate and divided into groups for RNA extraction, settlement, or further studies. Samples for RNA extraction were placed in a 2 ml Eppendorf tube and completely immersed in RNAlater (Thermofisher Scientific, USA) for extraction at a later date. Samples for settlement were added to 500 ml glass bowls containing settlement tiles (glass, ceramic tile, dead coral skeleton, and shell fragments) that were seasoned for 1–12 months in the field to induce rapid settlement [22]. Once skeleton formation could be seen by eye (3–5 days after settlement; Fig. 1d), individuals were carefully scraped from the surface using a scalpel and placed in RNAlater. A total of 10–25 swimming larvae or newly settled larvae were pooled for extraction to provide enough RNA for sequencing. All samples were from different individuals, except in 2014 where we collected both swimming and settled larvae from the same adult colony. A sample of adult tissue, taken from a single colony in April 2013, contained 4–5 polyps (less than 5 mm diameter plug), and a minimal amount of the underlying skeleton. The adult was different from those that were used to collect larvae. All adult colonies were returned to the reef after sampling and monitored for survivorship. Colony mortality was less than 5 %.

### Sample preparation and sequencing

Total RNA was isolated from swimming and recently settled larvae using the RNAqueous kit (Thermo Fisher Scientific, USA). The adult sample was homogenized directly into RNAqueous kit homogenization buffer and processed as per the manufacturer’s instructions. RNA was quantified, checked for quality with an agarose gel, and then sent to the Genomics Core Facility of the Research Technology Support Facilities of Michigan State University. Samples were quantified and quality checked using a BioAnalyzer (RNA integrity number > 8), and then converted into libraries using poly-A selection. Several methods were used to prepare and sequence samples (Table 1). For larval samples from 2012 (SRX1045048 and SRX1045047), the Illumina TruSeq mRNA version 2 library preparation kit was used. For adult samples from 2013 (SRX1045045 and SRX1045046), and larval samples from 2014 (SRX1045052, SRX1045051, SRX1045050, and SRX1045049; two runs per sample of swimming and settled larvae, respectively), the Illumina TruSeq Stranded mRNA library preparation kit LT was used. Libraries were pooled prior to sequencing. Samples from 2012 were sequenced using an Illumina HiSeq 2000 flow cell using TruSeq SBS version 3 reagents. For samples from 2013 and 2014, an Illumina HiSeq 2500 Rapid Run flow cell (v1) was used with Rapid SBS reagents. In 2012, larvae samples were multiplexed to run in one lane. The adult sample was sequenced in different runs on two lanes. Sequencing was done using 2 × 100 bp paired-end sequencing cycles producing 133 268 440, 109 623 674 and 104 806 800 read pairs from swimming larvae, settled larvae, and adult tissues, respectively. In 2014, another two samples from swimming and newly settled larvae were treated in the same way as the samples from 2013 but were sequenced with 2 × 150 bp paired-end sequencing cycles producing 25 183 766 and 20 628 911 read pairs, respectively. Base calling was done by Illumina Real Time Analysis (RTA) v1.17.21.3, and RTA output was demultiplexed and converted to FastQ format with Illumina Bcl2fastq v1.8.4.

**Table 1:** Summary of sample preparation

Source	N	Library	Sequencer	Cycles	# Reads
Swimming larvae	20 (2012), 12 (2014)	Illumina TruSeq mRNA Version 2 Library preparation Kit (2012); Illumina TruSeq Stranded mRNA Library preparation Kit LT (2014)	Illumina HiSeq 2000 flow cell using TruSeq SBS Version 3 reagents (2012); Illumina HiSeq 2500 Rapid Run flow cell (v1) with Rapid SBS reagents (2014)	2 × 100 bp paired-end (2012); 2 × 150 bp paired-end (2014)	133 268 440 (2012) and 25 183 766 (2014)
Newly settled larvae	15 (2012), 10 (2014)	Illumina TruSeq mRNA Version 2 Library preparation Kit (2012); Illumina TruSeq Stranded mRNA Library preparation Kit LT (2014)	Illumina HiSeq 2000 flow cell using TruSeq SBS Version 3 reagents (2012); Illumina HiSeq 2500 Rapid Run flow cell (v1) with Rapid SBS reagents (2014)	2 × 100 bp paired-end (2012); 2 × 150 bp paired-end (2014)	109 623 674 (2012) and 20 628 911 (2014)
Adult	1	Illumina TruSeq Stranded mRNA Library preparation Kit LT (2013)	Illumina HiSeq 2500 Rapid Run flow cell (v1) with Rapid SBS reagents	2 × 100 bp paired-end	104 806 800

### Transcriptome preprocessing, assembly and annotation

Sequencing data from all five samples (393 511 591 paired-end reads) were pooled for assembly. Data went through two stages of read trimming: first, we performed quality-based trimming using Trimmomatic v0.33 [7]. A sliding window of 4 bp and trimming threshold of phred score equal to 2 were chosen to maximize sensitivity [23], followed by K-mer spectral analysis to remove low abundance k-mers using ‘filter-abund.py –V’ from the Khmer 2.0 package [24]. FastQC v0.11.3 was used to check data quality before and after trimming [25]. After filtering, the remaining 391 297 779 high-quality read pairs were used for *de novo* transcriptome assembly using Trinity v6.0.2 producing 881 402 transcripts [26]. SeqClean was used to trim poly-A tails and remove low complexity sequences [27]. To enhance the quality of the assembly, 1077 short transcripts (<200 bp) were excluded. To exclude 11 380 uncovered isoforms, we also back-mapped input sequencing reads to the assembly using Salmon software, which allows an unambiguous alignment [28]. For functional annotation, assembled transcripts were blasted against the Swiss-Prot database and best hits with *p*-values less than 1 × e10^−3^ were selected. Assembly and annotation statistics are listed in Table 2.

**Table 2:** Transcriptome assembly and annotation statistics compared with previous *P. astreoides* transcriptome

Assembly components	*P. astreoides* + *Symbiodinium* sp. 2016	*P. astreoides* alone 2016	*P. astreoides* alone 2013 [3]	*Symbiodinium* sp. alone 2016
Genes	717 454	95 294	29 422	145 570
Transcripts	867 255	129 718	30 740	186 177
GC %	44	40	42	48
Contig N10	3876	4642	1574	5230
Contig N20	2568	3321	1207	3701
Contig N30	1871	2487	949	2825
Contig N40	1386	1867	767	2236
Contig N50	1005	1358	661	1804
n under 200	0	0	2164	0
n over 1 k	145 836	27 334	3274	67 354
n over 10 k	319	58	0	260
Median contig length (bp)	389	429	418	659
Average contig length (bp)	685	811	550	1105
Maximum length (bp)	28 297	19 877	8171	28 297
Minimum length (bp)	201	201	100	201
Bases Ns	0	0	924	0
Total assembled bases	594 399 145	105 218 865	16 907 062	205 741 294
N50 (longest transcript per unigene)	763	967	640	1623
Total assembled bases (longest transcript per unigene)	430 507 117	65 529 888	15 807 055	145 440 520
No. of possible ORF	578 372	49 348	18 351	221 299
No. of transcripts with ORF	327 829	33 331	15 183	89 425
No. of all possible complete ORF	223 741	26 802	3666	124 810
No. of transcripts with complete ORF	123 339	17 277	2932	59 126
No. of transcripts with Swiss-Prot blast hit	204 071	25 384	15 492	52 308

To identify zooxanthellae transcripts, the assembled sequences were compared against a homemade library of publically available zooxanthellae transcriptomes using BLASTN for sequences with e-values less than 10^−5^. The library included transcriptomes for *Symbiodinium* clades A and B [29], *S. minutum* [30], *S. kawagutii*, *Symbiodinium* sp. (Clades C1, C15, CCMP2430, and Mp), *Alexandrium fundyense*, *A. monilatum*, *A. temarense*, *Peridinium aciculiferum*, *Karenia brevis* (CCMP2229, SP1, SP3, and Wilson), and *Prorocentrum minimum* (CCMP1329 and CCMP2233) [31]. To identify a high confidence *P. astreoides* transcriptome, the zooxanthellae-free transcripts went through a second BLASTN screen using two homemade libraries of publically available Cnidarian transcriptomes and genomic sequences for sequences with e-values less than 10^−5^. The Cnidarian transcriptome library included *Acropora digitifera* [32], *A. millepora* [33], *A. hyacinthus*, *A. tenuis* [34], *P. astreoides* [3], *Nematostella vectensis* [35], and *Hydra vulgaris* [25]. The genomic library included the genomes of *A. digitifera* [32] and *N. vectensis* [35], in addition to genomic sequences from the Trace archive [26] for *A. millepora*, *A. palmata*, *P. lobata*, and *M. faveolata*.

To assess the differences between our *P. astreoides* transcriptome and the previously published one [3], Transrate (v1.0.1) [27] was used to examine the contigs of both assemblies. We used Transrate to run a conditional reciprocal best BLAST analysis using the older, smaller assembly as a query against our new assembly as a reference [36]. A *p*-value of 1x10^−5^ was used as a threshold. Then, we compared the change of transcript length between reciprocal hits and calculated the total gain or loss in the transcript lengths. The conditional reciprocal BLAST using the original assembly (30 740 isoforms) as a query against our assembly as a reference was more sensitive than the classical reciprocal BLAST. This identified 21 232 reciprocal hits (17 382 of which were unique), and resulted in a total gain of approximately 30 Mbp and a loss of 2.5 Mbp in the transcript lengths.

### Availability of supporting data

The raw data used for assembly are deposited into the National Center for Biotechnology Information (NCBI) Sequence Reads Archive (SRA) under accession number SRX1045045-52, which is associated with BioProject number PRJNA283441. This Transcriptome Shotgun Assembly project has been deposited at DDBJ/ENA/GenBank under the accession GEHP0000000. Further supporting data can be found in the *GigaScience* repository, GigaDB [37].

## Abbreviations

DRNA, Departamento de Recursos Naturales y Ambientales (Department of Natural and Environmental Resources); NCBI, National Center for Biotechnology Information; RTA, real time analysis; SRA, Sequence Read Archive
