# Correction to: Transcriptome of the caribbean stony coral *Porites astreoides* from three developmental stages

**DOI:** 10.1093/gigascience/giz048

**Published:** 2019-11-15

**Authors:** Tamer A Mansour, Joshua J C Rosenthal, C Titus Brown, Loretta M Roberson

**Affiliations:** 1 Department of Population Health and Reproduction, University of California, Davis, California, USA; 2 Department of Clinical Pathology, College of Medicine, Mansoura University, Mansoura, Egypt; 3 Marine Biological Laboratory, Woods Hole, Massachusetts, USA; 4 Department of Environmental Science, University of Puerto Rico Río Piedras, San Juan, Puerto Rico, USA; 5 Institute of Neurobiology, University of Puerto Rico Medical Sciences Campus, San Juan, Puerto Rico, USA

This is a correction to: *GigaScience*, Volume 5, Issue 1, 1 December 2016, s13742-016-0138-1, https://doi.org/10.1186/s13742-016-0138-1

In the original version of the article “Transcriptome of the Caribbean stony coral *Porites astreoides* from three developmental stages” by Tamer A. Mansour et al. [1] there were a number of unfortunate errors in the text caused due to problems with citation management software. In the paragraph titled “Transcriptome preprocessing, assembly and annotation”, the order of references 7, 25, 26, 27, 36 are incorrect, and the Trinity version is also wrong. Furthermore, reference 14 [2] was misspelled, five references were missed (22 [3], 25 [4], 26 [5], 27 [6] and 36 [7]) and subsequently incorrectly ordered (references 37 [8], 38 [9] and 39 [10]). The references and the Trinity version have been corrected, and the authors apologize for the error.
